# Effect of Body Mass Index and Intra-Abdominal Fat Measured by Computed Tomography on the Risk of Bowel Symptoms

**DOI:** 10.1371/journal.pone.0123993

**Published:** 2015-04-23

**Authors:** Naoyoshi Nagata, Kayo Sakamoto, Tomohiro Arai, Ryota Niikura, Takuro Shimbo, Masafumi Shinozaki, Noriko Ihana, Katsunori Sekine, Hidetaka Okubo, Kazuhiro Watanabe, Toshiyuki Sakurai, Chizu Yokoi, Mikio Yanase, Junichi Akiyama, Naomi Uemura, Mitsuhiko Noda

**Affiliations:** 1 Department of Gastroenterology and Hepatology, National Center for Global Health and Medicine, 1-21-1, Toyama, Shinjuku-ku, Tokyo, 162–8655, Japan; 2 Department of Diagnostic Radiology, National Center for Global Health and Medicine, 1-21-1, Toyama, Shinjuku-ku, Tokyo, 162–8655, Japan; 3 Department of Clinical Research and Informatics, International Clinical Research Center Research Institute, National Center for Global Health and Medicine, 1-21-1, Toyama, Shinjuku-ku, Tokyo, 162–8655, Japan; 4 Department of Gastroenterology and Hepatology, National Center for Global Health and Medicine, Kohnodai Hospital, 1-7-1, Kohnodai, Ichikawa, Chiba, 272–8516, Japan; 5 Diabetes Research, Diabetes Research Center, National Center for Global Health and Medicine, 1-21-1, Toyama, Shinjuku-ku, Tokyo, 162–8655, Japan; Faculty of Biology, SPAIN

## Abstract

**Background:**

This study aims to investigate the association between body mass index (BMI) or intra-abdominal fat measured by computed tomography (CT) and bowel symptoms.

**Method:**

A cohort of 958 Japanese adults who underwent colonoscopy and CT and completed questionnaires after excluding colorectal diseases was analyzed. Six symptoms (constipation, diarrhea, loose stools, hard stools, fecal urgency, and incomplete evacuation) using a 7-point Likert scale were evaluated between baseline and second questionnaire for test-retest reliability. Associations between BMI, visceral adipose tissue (VAT), subcutaneous adipose tissue (SAT), and symptom score were analyzed by a rank-ordered logistic model, adjusting for age, sex, smoking, and alcohol consumption, hypertension, diabetes mellitus, and dyslipidemia.

**Results:**

Some bowel symptom scores were significantly (p<0.05) different between the age groups, sexes, smoking, and alcohol consumption. In multivariate analysis, constipation was associated with low BMI (p<0.01), low VAT area (p = 0.01), and low SAT area (p<0.01). Moreover, hard stools was associated with low BMI (p<0.01) and low SAT area (p<0.01). The remaining symptoms were not significantly associated with BMI or intra-abdominal fat. Test-retest reliability of bowel symptom scores with a mean duration of 7.5 months was good (mean kappa, 0.672).

**Conclusions:**

Both low BMI and low abdominal fat accumulation appears to be useful indicators of increased risk for constipation and hard stools. The long-term test-retest reliability of symptom score suggests that bowel symptoms relevant to BMI or visceral fat remain consistent over several months.

## Introduction

Gastrointestinal (GI) symptoms are common in the general population, but studies on the role of GI symptoms in overweight individuals are limited.[1,2] Several studies have reported an increased risk of upper GI symptoms associated with high body mass index (BMI);[3–6] however, little information is available on the association between high BMI and functional bowel disorder.[4,6,7] Recent studies have shown that abdominal visceral fat as measured by computed tomography (CT) is a better predictor of the risk of upper GI disease (e.g., gastroesophageal reflux disease or Barrett’s esophagus) than BMI.[8,9] However, the association between lower GI symptoms and CT-evaluated intra-abdominal fat has not been reported previously.

This study examined the relationship of BMI, CT-measured visceral adipose tissue (VAT), and CT-measured subcutaneous adipose tissue (SAT) with the risk of bowel symptoms. Bowel symptoms are caused by various colorectal diseases; however, previous population-based studies have not excluded colorectal diseases.[10–12] We therefore used colonoscopy to exclude the presence of colorectal diseases and applying the criteria of functional bowel disorder.[13]

## Methods

### Study Design, Setting, and Participants

**Fig 1:**
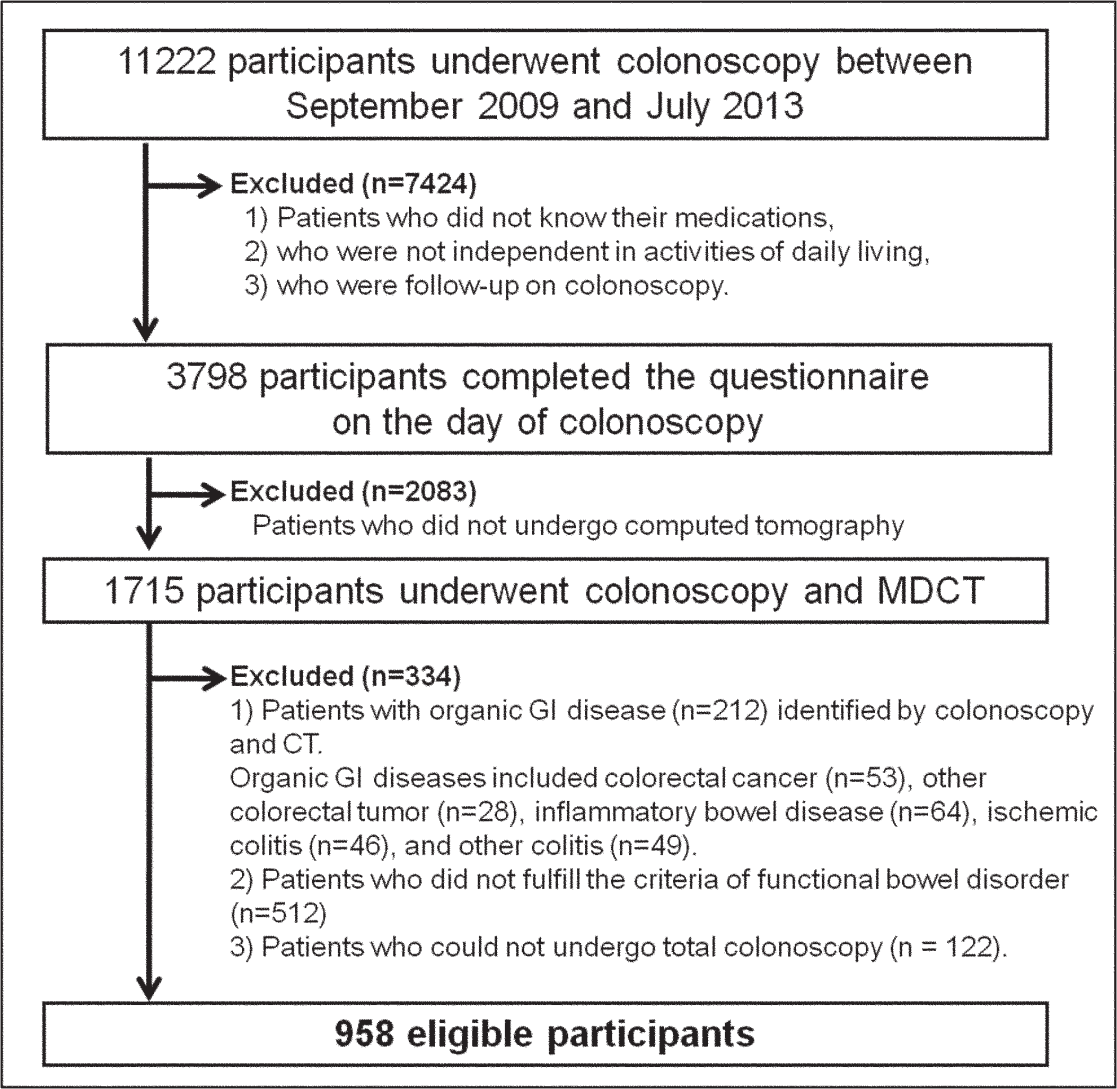
Study flow. Abbreviations: MDCT, multidetector computed tomography; GI, gastrointestinal.

We conducted a prospective, hospital-based, cross-sectional study of Japanese adults at the National Center for Global Health and Medicine (NCGM), Japan between September 2009 and June 2013. Participants who underwent elective colonoscopy and CT and completed a questionnaire were enrolled. The patient population and data collection procedure are described in detail in our previous report of a study with different research objectives from the present study [14]. We included patients > 18 years of age presenting with bowel symptoms or requiring colorectal cancer screening. Patients were excluded if their medication use was unknown, they were not independent in activities of daily living, or they were being followed after colonoscopy. Of the 11,222 eligible participants, 3798 Japanese patients completed the questionnaire (Fig 1), of which 1715 underwent multidetector computed tomography (MDCT) before colonoscopy. At our institution, MDCT is performed for individuals who request screening for non-colorectal cancer (e.g., stomach, pancreas, liver, biliary tract, lung, prostate, uterus, or ovarian cancer) or for the investigation of rectal bleeding, diarrhea, and abdominal pain in patients presenting to our institution’s emergency outpatient department. The remaining 2083 participants who did not undergo MDCT (1393 men; mean age, 64.0 years) declined either because of anxiety due to radiation exposure or cost or because they had previously undergone CT at another institution. From the 1715 patients who underwent colonoscopy and MDCT, we excluded the following patients: (i) those with colorectal diseases identified by colonoscopy and CT (n = 212); (ii) those who did not fulfill the criteria for functional bowel disorder[13] (n = 512); and (iii) those who could not undergo total colonoscopy (n = 122). This left 958 patients for analysis in this study.

This study was approved by the ethics committee of the National Center for Global Health and Medicine Center (No. 1012) and was implemented in accordance with the provisions of the Declaration of Helsinki. Written informed consent was obtained from all patients prior to endoscopy and CT.

### Evaluation of Bowel Symptoms

Diagnosis of functional bowel disorder requires the existence of certain characteristic bowel symptoms over the past 3 months, with an onset of more than 6 months before colonoscopy.[13] On day of pre-colonoscopy, bowel symptoms were evaluated using the GI symptom rating scale (GSRS), which covered 6 bowel symptoms using a 7-point Likert scale (1, none at all; 2, minor; 3, mild; 4, moderate; 5, moderately severe; 6, severe; and 7, very severe).[15,16] The validity of GSRS has been well documented in conditions of functional bowel disorder.[17–19] The 6 bowel symptoms included constipation, diarrhea, loose stools, hard stools, fecal urgency, and incomplete evacuation.

To assess the test-retest reliability of bowel symptoms scores, we conducted a secondary questionnaire using the same GSRS among participants who visited our department over 1 week after the first interview.

### Diagnosis of colorectal diseases

An electronic high-resolution video endoscope (model CFH260; Olympus Optical, Tokyo, Japan) with full preparation was used for diagnosis of colorectal diseases. Endoscopy was performed by well-trained staff who were blinded to the questionnaire results. When abnormalities were detected by colonoscopy, biopsy, polypectomy, or endoscopic mucosal resection was performed. All removed specimens were evaluated by expert pathologists, and final diagnoses of colorectal diseases were made.

Colorectal diseases included early and advanced colorectal cancer, other colorectal tumor, inflammatory bowel disease, ischemic colitis, and other colitis, as reported previously.[20] During the same period (within 1 week of colonoscopy), upper endoscopy was performed for 110 patients, none of whom were found to have cancerous or ulcerous lesions or severe gastritis.

### Exposure Variables

A detailed questionnaire was completed at the endoscopy unit on the same day prior to colonoscopy.[14] Patients were asked about alcohol consumption and smoking status, and their medical history was recorded by well-trained medical researchers. Researchers also checked prescriptions and medical records in addition to the information provided by the patients to avoid inadvertences. Medical history included diseases such as hypertension, diabetes mellitus, and dyslipidemia, which were considered present in patients taking disease-specific drugs. Smoking status was classified as current (daily or occasionally) smoker, former smoker, or never smoker. BMI was calculated as weight divided by height squared (kg/m^2^).

### Measurement of the Abdominal Adipose Tissue Area by multidetector CT

**Fig 2:**
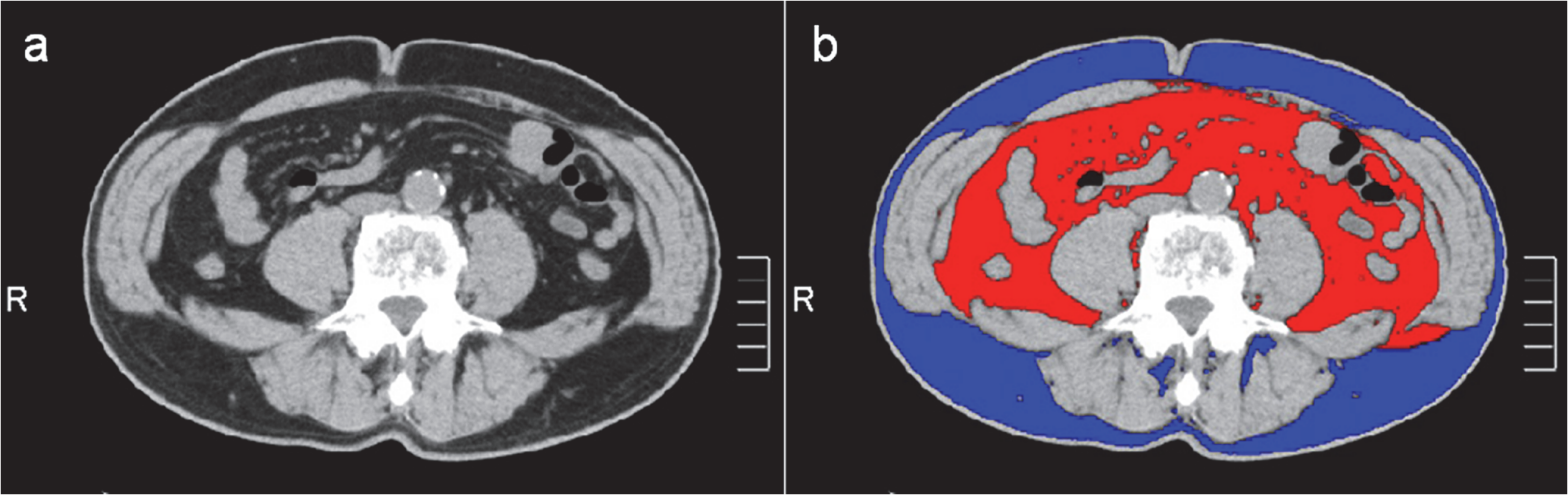
Measurement of intra-abdominal fat area by use of multidetector CT. A 61-year-old-male with a BMI of 24.8. (a) Initial CT images. (b) Intra-abdominal adipose tissue areas. Regions of red and blue color indicate visceral (107.3 cm^2^) and subcutaneous (104.1 cm^2^) adipose tissue, respectively.

The technique used for measuring the adipose tissue area on CT has been standardized and validated,[21] and shows only negligible inter-observer variation.[22] Participants were assessed in the supine position using a 320-row area detector CT scanner (Aquilion ONE, Toshiba Medical Systems, Japan). All CT examinations were performed with helical scanning using the following parameters: 64 × 0.5 mm collimation, 120 kVp; auto exposure control (AEC) beam pitch, 0.83 (table feed per gantry, 53 mm; collimation beam width, 64 mm); gantry rotation time, 0.5 s; matrix, 512 × 512; and field of view, 350–500 mm. All images were reconstructed using a standard reconstruction algorithm with a section thickness of 5 mm. The cross-sectional surface areas (cm^2^) of different abdominal fat compartments were calculated at this slice using commercially available CT software (Aquilion ONE, Toshiba Medical Systems) to determine the adipose tissue area electronically by setting the attenuation values for a region of interest within the range of -150 to -30 Hounsfield units. The VAT area was defined as intra-abdominal fat bound by the parietal peritoneum or transversalis fascia, excluding the vertebral column and paraspinal muscles (**Fig 2**). The SAT area was defined as fat superficial to the abdominal and back muscles (**Fig 2**). A region of interest drawn around the external margin of the dermis was used to calculate the total adipose tissue area. The SAT area was calculated by subtracting the VAT area from the total adipose tissue area.

### Statistical Analysis

GSRS scores were compared using the Mann–Whitney U test. Rank-ordered logistic model[23] was used to determine the association between bowel symptoms and BMI, VAT area, or SAT area. Multivariate analysis was adjusted for age, sex,[20] alcohol consumption,[24] smoking status,[20,25] and diabetes mellitus,[26] all of which are known factors associated with functional bowel disorder.[13] Dyslipidemia and hypertension were potential confounders between obesity and bowel symptoms; we therefore also included these in the multivariate model. These associations were evaluated after excluding colorectal diseases on colonoscopy.

The test-retest reliability of the bowel symptom scores in the GSRS from the first and second questionnaires was analyzed using kappa statistics. Kappa values >0.80 denoted excellent agreement, >0.60–0.80 good,> 0.40–0.60 moderate, >0.20–0.40 fair, and ≤0.20 poor.[27] P<0.05 was considered significant. All statistical analysis was performed using Stata version 13 software (StataCorp, College Station, TX).

## Results

### Patient Characteristics

Baseline characteristics are shown in **Table 1**. Of the 958 patients who underwent colonoscopy plus CT and completed questionnaires, 441 (46%) were aged ≥65 years, and 647 (68%) were male. The mean BMI was 22.8 kg/m^2^and 244 patients (26%) had a BMI of ≥25 kg/m^2^.

**Table 1:** Characteristics of the 958 patients included in the study.

Age (years)	60.7 ± 14.5
Age ≥65 (years)	441 (46.0)
Male sex	647 (67.5)
Current smoker	232 (24.2)
Former smoker	269 (28.1)
Never smoker	457 (47.7)
Current drinker	516 (53.9)
Hypertension	340 (35.5)
Diabetes mellitus	158 (16.5)
Dyslipidemia	167 (17.4)
Height	163.2 ± 9.5
Weight	61.0 ± 12.8
Body mass index, kg/m^2^	22.8 ± 3.8
< 18.5	107 (11.2)
18.5–24.9	607 (63.4)
25–29.9	206 (21.5)
30–34.9	32 (3.3)
35–39.9	5 (0.5)
≥ 40	1 (0.1)
Visceral adipose tissue area, cm^2^	112.5 ± 82.6
Subcutaneous adipose tissue area, cm^2^	124.8 ± 74.4
Visceral adipose tissue to subcutaneous adipose tissue ratio	1.02 ± 0.75
Total adipose tissue area, cm^2^	234.8 ± 128.0
**Bowel symptom scores (range)**	
Constipation (1–7)	2.0 ± 1.5
Diarrhea (1–7)	2.1 ± 1.5
Loose stools (1–7)	1.9 ± 1.3
Hard stools (1–7)	1.9 ± 1.3
Fecal urgency (1–7)	2.1 ± 1.5
Incomplete evacuation (1–7)	2.3 ± 1.3

Values presented with plus/minus sign represent mean ± standard deviation.

### Patient Characteristics and Bowel Symptoms

The association of patient characteristics and 6 bowel symptom scores is shown in **Table 2**. Among the age groups (age ≥65 and <65 years), patients aged <65 years had higher symptom scores for diarrhea, loose stools, fecal urgency, and incomplete evacuation than those aged ≥65 years. Among the 2 sexes, males had higher symptom scores for diarrhea and loose stools, whereas females had higher symptom scores for constipation and hard stools. Smokers had higher symptom scores for constipation, diarrhea, loose stools, and fecal urgency than non-smokers. Drinkers had lower symptom scores for constipation, diarrhea, loose stools, and fecal urgency than non-drinkers. Between patients with and without hypertension, no significant difference was noted in any of the 6 bowel symptoms. Patients with diabetes had higher symptom scores for hard stools than patients without diabetes.

**Table 2:** Association of patient characteristics and bowel symptom scores (n = 958).

	Age ≥65/<65	p	Male/female	p	Current smoking (yes/no)	p	Alcohol consumption (yes/no)	p	Hypertension (with/without)	p	Diabetes mellitus (with/without)	p	Dyslipidemia (with/without)	p
Constipation	2.0 ± 1.4/ 2.0 ± 1.5	0.795	1.9 ± 1.4/ 2.2 ± 1.5	**<0.01**	2.2 ± 1.6/ 2.0 ± 1.4	**0.04**	2.0 ± 1.5/ 2.1 ± 1.5	**0.04**	2.0 ± 1.5/ 2.0 ± 1.5	0.35	2.2 ± 1.6/ 2.0 ± 1.4	0.48	2.0 ± 1.4/ 2.0 ± 1.5	0.81
Diarrhea	1.8 ± 1.3/ 2.3 ± 1.6	**<0.01**	2.1 ± 1.5 /1.9 ± 1.5	**<0.01**	2.3 ± 1.6/ 2.0 ± 1.5	**0.02**	2.0 ± 1.4/ 2.1 ± 1.6	**0.02**	2.0 ± 1.4/ 2.1 ± 1.6	0.40	2.0 ± 1.4/ 2.1 ± 1.5	0.92	1.9 ± 1.3/ 2.1 ± 1.5	0.07
Loose stools	1.7 ± 1.1/ 2.1 ± 1.4	**<0.01**	2.0 ± 1.3/ 1.8 ± 1.2	**<0.01**	2.1 ± 1.4/ 1.9 ± 1.3	**0.03**	1.9 ± 1.2/ 1.9 ± 1.3	**0.03**	1.8 ± 1.2/ 2.0 ± 1.4	0.16	1.9 ± 1.1/ 1.9 ± 1.3	0.67	1.9 ± 1.2/ 1.9 ± 1.3	0.91
Hard stools	1.9 ± 1.3/ 1.9 ± 1.3	0.579	1.8 ± 1.2/ 2.1 ± 1.4	**0.01**	1.9 ± 1.3/ 1.9 ± 1.3	0.85	1.9 ± 1.2/ 1.9 ± 1.3	0.85	1.9 ± 1.2/ 2.0 ± 1.3	0.20	2.2 ± 1.5/ 1.9 ± 1.2	**0.02**	2.0 ± 1.4/ 1.9 ± 1.3	0.28
Fecal urgency	1.9 ± 1.3/ 2.3 ± 1.6	**<0.01**	2.1 ± 1.5/ 2.0 ± 1.4	0.15	2.3 ± 1.6/ 2.0 ± 1.4	**<0.01**	2.1 ± 1.4/ 2.1 ± 1.5	**<0.01**	2.1 ± 1.5/ 2.1 ± 1.4	0.41	2.0 ± 1.4/ 2.1 ± 1.5	0.71	2.0 ± 1.4/ 2.1 ± 1.5	0.74
Incomplete evacuation	2.1 ± 1.3/ 2.4 ± 1.4	**<0.01**	2.3 ± 1.4/ 2.2 ± 1.3	0.26	2.4 ± 1.5/ 2.2 ± 1.3	0.63	2.2 ± 1.3/ 2.3 ± 1.4	0.63	2.1 ± 1.3/ 2.3 ± 1.4	0.06	2.3 ± 1.3/ 2.3 ± 1.3	0.40	2.2 ± 1.2/ 2.3 ± 1.4	0.63

Values presented with plus/minus sign represent mean ± standard deviation. Differences in symptom scores between 2 groups were analyzed using the Mann–Whitney U test. *p-values of <0.05 represent comparison of patients with and without the corresponding characteristics.

### Associations between Obesity Index and Bowel Symptoms

The association of BMI and intra-abdominal fat and bowel symptoms is shown in **Table 3**. Rank-ordered logistic model revealed that constipation was associated with low BMI (p < 0.01), low VAT area (p = 0.01), and low SAT area (p < 0.01), while hard stools was associated with low BMI (p < 0.01) and low SAT area (p < 0.01) in multivariate analysis. However, diarrhea, loose stools, fecal urgency, and incomplete evacuation were not associated with BMI or intra-abdominal fat.

**Table 3:** Effect of body mass index, visceral adipose tissue, and subcutaneous adipose tissue on the risk of bowel symptoms (n = 958).

	Univariate coefficient	p-value	Multivariate coefficient*	p-value
**Constipation**				
Body mass index	−0.0588	**<0.01**	−0.0537	**<0.01**
Visceral adipose tissue area	−0.0026	**<0.01**	−0.0024	**0.01**
Subcutaneous adipose tissue area	−0.0027	**<0.01**	−0.0032	**<0.01**
**Diarrhea**				
Body mass index	−0.0191	0.25	−0.0181	0.30
Visceral adipose tissue area	−0.0005	0.51	0.0001	0.90
Subcutaneous adipose tissue area	−0.0019	**0.03**	−0.0007	0.41
**Loose stools**				
Body mass index	−0.0167	0.31	−0.0236	0.18
Visceral adipose tissue area	−0.0015	0. 07	−0.0013	0.11
Subcutaneous adipose tissue area	−0.0014	0.09	−0.0008	0.39
**Hard stools**				
Body mass index	−0.0422	**0.01**	−0.0481	**<0.01**
Visceral adipose tissue area	−0.0009	0.26	−0.0006	0.47
Subcutaneous adipose tissue area	−0.0018	**0.04**	−0.0026	**<0.01**
**Fecal urgency**				
Body mass index	−0.0292	0.08	−0.0312	0.08
Visceral adipose tissue area	−0.0011	0.15	−0.0008	0.32
Subcutaneous adipose tissue area	−0.0015	0.07	−0.0010	0.28
**Incomplete evacuation**				
Body mass index	−0.0260	0.10	−0.0224	0.18
Visceral adipose tissue area	−0.0009	0.24	−0.0005	0.57
Subcutaneous adipose tissue area	−0.0014	0.08	−0.0009	0.31

*Adjusted for age, sex, current smoking, alcohol consumption, hypertension, diabetes mellitus, and dyslipidemia.

### Reliability of bowel symptom score

Among the 958 participants, 690 (72.0%) completed a secondary questionnaire of using the GSRS within a mean duration of 7.5±10.7 months. The test-retest reliability of the GSRS was good (mean Kappa values was 0.672; **Table 4**).

**Table 4:** Test-retest reliability of bowel symptom score between 1st and 2nd questionnaire (n = 690).

	Agreement	Kappa	SE	P
Constipation	79.0%	0.686	0.022	<0.001
Diarrhea	80.1%	0.691	0.022	<0.001
Loose stools	79.1%	0.675	0.024	<0.001
Hard stools	78.1%	0.667	0.023	<0.001
Fecal urgency	76.7%	0.656	0.022	<0.001
Incomplete evacuation	74.9%	0.657	0.021	<0.001

Abbreviations: GI, Gastrointestinal: SE, standard error.

## Discussion

To the best of our knowledge, this is the first study evaluating the relationship between functional bowel symptoms and BMI, VAT area, and SAT area as calculated by CT. After excluding colorectal diseases using colonoscopy and applying the criteria of functional bowel disorder, we found that constipation was associated with low BMI, low VAT area, and low SAT area, while hard stools was associated with low BMI and low SAT area. Finally we found that long-term test re-test reliability of GSRS was good.

In previous studies, the relationship between bowel symptoms and BMI has been controversial. For example, a cross-sectional study of 16,078 participants from China demonstrated that high BMI was associated with functional diarrhea.[28] A case-control study of 96 IBS patients from Sweden identified high BMI to be associated with the severity of bloating, gas, urgency, loose stools, and stool frequency.[29] In contrast, a well-designed birth cohort study of young adults in Australia showed that BMI was not associated with IBS, including bloating and constipation.[30] In a Korean study of 5,605 participants who took part in a health screening program, BMI and waist circumference were not risk factors of IBS after excluding organic disease.[20] Among 2,712 Japanese who underwent health check-ups, BMI was not associated with increased risk of any type of IBS.[25] In another study of 2,495 Japanese subjects who underwent health check-ups, BMI did not differ among groups of non-IBS, patients with constipation type-IBS, or patients with diarrhea type-IBD. Although different results were observed on account of the differences in study design, sample size, and ethics, diarrhea symptoms appear to be positively associated with obesity, particularly in Western countries. However, in Asian studies including the present study, no relationship between bowel symptoms and high BMI has been identified.

A few studies have investigated the relationship between low BMI and bowel symptoms. Kubo et al.[25] reported that low BMI (OR: 0.95) was significantly associated with IBS in multivariate analysis. Farzaneh et al.[31] identified low BMI (OR: 0.94) as an independent risk factor associated with IBS in Iran. These 2 Asian studies support our findings.

Reproducible relates to the interpretation of scores from psychometric instruments (eg, symptom scales, questionnaires, and observer ratings) used in clinical practice. In this study, we confirmed a good long-term test-retest reliability (mean kappa, 0.66), which is above the kappa value of 0.6 usually considered to be good.[32] Our results imply that patients with specific GI symptoms and the severity of these symptoms remain consistent over several months.

This study has several strengths. First, colonoscopy enables the exclusion of colorectal diseases. Second, we were able to confirm the long-term reliability of the bowel symptom scores. Third, the sample size was relatively large, facilitating adjustment for confounding factors. However, this study also has limitations. First, although, our subjects met the definition of functional bowel disorder, we could not classify functional bowel disorder into IBS, functional bloating, functional constipation, functional diarrhea, and unspecified functional bowel disorder because we did not assess stool condition, such as improvement with defecation, onset associated with a change in stool frequency, or onset associated with a change in stool form.[13] Second, although we collected data on smoking and alcohol consumption habits and metabolic factors while interviewing patients in the pre-colonoscopy setting in the endoscopy room, we did not obtain information on educational level or somatic disorders that may be associated with functional bowel disorder.[13] In particular, somatic disorders, which are characterized by somatic symptoms (e.g., pain, GI distress, and sexual problems) and pseudoneurological symptoms (e.g., amnesia and breathing difficulties), tend to be accompanied by high levels of worry, anxiety, and increased reactions in response to physical symptoms[33] and to show a positive association with bowel symptoms.[34] Thus, the lack of assessment of somatic disorder in this study is a major limitation. Third, we did not evaluate the composition of feces (bacteria, fat, or pH), conduct lactose intolerance tests, or perform breath tests to rule out small intestinal bacterial overgrowth in this study, which are all useful examinations for diagnosing functional bowel disorder.[35–37] Fourth, both bowel symptoms and BMI or intra-abdominal fat was associated with dietary intake or dietary pattern[38], but we could not the diet information. In the pre-colonoscopy setting in the endoscopic room, we could gather information only on lifestyle habits, medication use, comorbidities, and bowel symptoms scores.

In conclusion, both low BMI and low abdominal fat accumulation increased risk of constipation and hard stools. The long-term test-retest reliability of symptom score suggests that bowel symptoms relevant to BMI or visceral fat remain consistent over several months.

## References

[1] Moayyedi P. The epidemiology of obesity and gastrointestinal and other diseases: An overview. Dig Dis Sci. 2008;53: 2293–2299. 10.1007/s10620-008-0410-z 18636328

[2] Eslick GD. Gastrointestinal symptoms and obesity: A meta-analysis. Obes Rev. 2012;13: 469–479. 10.1111/j.1467-789X.2011.00969.x 22188520

[3] Aro P, Ronkainen J, Talley NJ, Storskrubb T, Bolling-Sternevald E, Agreus L. Body mass index and chronic unexplained gastrointestinal symptoms: An adult endoscopic population based study. Gut. 2005;54: 1377–1383. 15917313 10.1136/gut.2004.057497 PMC1774688

[4] Delgado-Aros S, Locke GR 3rd, Camilleri M, Talley NJ, Fett S, Zinsmeister AR, et al Obesity is associated with increased risk of gastrointestinal symptoms: A population-based study. Am J Gastroenterol. 2004;99: 1801–1806. 15330922 10.1111/j.1572-0241.2004.30887.x

[5] El-Serag HB, Graham DY, Satia JA, Rabeneck L. Obesity is an independent risk factor for GERD symptoms and erosive esophagitis. Am J Gastroenterol. 2005;100: 1243–1250. 15929752 10.1111/j.1572-0241.2005.41703.x

[6] Crowell MD, Cheskin LJ, Musial F. Prevalence of gastrointestinal symptoms in obese and normal weight binge eaters. Am J Gastroenterol. 1994;89: 387–391. 8122651

[7] Talley NJ, Quan C, Jones MP, Horowitz M. Association of upper and lower gastrointestinal tract symptoms with body mass index in an apanesen cohort. Neurogastroenterol Motil. 2004;16: 413–419. 15305996 10.1111/j.1365-2982.2004.00530.x

[8] El-Serag HB, Hashmi A, Garcia J, Richardson P, Alsarraj A, Fitzgerald S, et al Visceral abdominal obesity measured by CT scan is associated with an increased risk of barrett’s oesophagus: A case-control study. Gut. 2014;63: 220–229. 10.1136/gutjnl-2012-304189 23408348 PMC3976427

[9] Nam SY, Choi IJ, Ryu KH, Park BJ, Kim HB, Nam BH. Abdominal visceral adipose tissue volume is associated with increased risk of erosive esophagitis in men and women. Gastroenterology. 2010;139: 1902–1911.e2. 10.1053/j.gastro.2010.08.019 20727886

[10] Andrews EB, Eaton SC, Hollis KA, Hopkins JS, Ameen V, Hamm LR, et al Prevalence and demographics of irritable bowel syndrome: Results from a large web-based survey. Aliment Pharmacol Ther. 2005;22: 935–942. 16268967 10.1111/j.1365-2036.2005.02671.x

[11] Lee SY, Lee KJ, Kim SJ, Cho SW. Prevalence and risk factors for overlaps between gastroesophageal reflux disease, dyspepsia, and irritable bowel syndrome: A population-based study. Digestion. 2009;79: 196–201. 10.1159/000211715 19342860

[12] Han SH, Lee OY, Bae SC, Lee SH, Chang YK, Yang SY, et al Prevalence of irritable bowel syndrome in korea: Population-based survey using the rome II criteria. J Gastroenterol Hepatol. 2006;21: 1687–1692. 16984590 10.1111/j.1440-1746.2006.04269.x

[13] Longstreth GF, Thompson WG, Chey WD, Houghton LA, Mearin F, Spiller RC. Functional bowel disorders. Gastroenterology. 2006;130: 1480–1491. 16678561 10.1053/j.gastro.2005.11.061

[14] Nagata N, Sakamoto K, Arai T, Niikura R, Shimbo T, Shinozaki M, et al. Visceral abdominal fat measured by computed tomography is associated with an increased risk of colorectal adenoma. Int J Cancer. 2014. 10.1002/ijc.28872 24692064

[15] Svedlund J, Sjodin I, Dotevall G. GSRS—a clinical rating scale for gastrointestinal symptoms in patients with irritable bowel syndrome and peptic ulcer disease. Dig Dis Sci. 1988;33: 129–134. 3123181 10.1007/BF01535722

[16] Wiklund IK, Fullerton S, Hawkey CJ, Jones RH, Longstreth GF, Mayer EA, et al An irritable bowel syndrome-specific symptom questionnaire: Development and validation. Scand J Gastroenterol. 2003;38: 947–954. 14531531 10.1080/00365520310004209

[17] Dimenas E, Glise H, Hallerback B, Hernqvist H, Svedlund J, Wiklund I. Well-being and gastrointestinal symptoms among patients referred to endoscopy owing to suspected duodenal ulcer. Scand J Gastroenterol. 1995;30: 1046–1052. 8578162 10.3109/00365529509101605

[18] Shiotani A, Miyanishi T, Takahashi T. Sex differences in irritable bowel syndrome in apanese university students. J Gastroenterol. 2006;41: 562–568. 16868804 10.1007/s00535-006-1805-2

[19] Engsbro AL, Begtrup LM, Kjeldsen J, Larsen PV, de Muckadell OS, Jarbol DE, et al Patients suspected of irritable bowel syndrome—cross-sectional study exploring the sensitivity of rome III criteria in primary care. Am J Gastroenterol. 2013;108: 972–980. 10.1038/ajg.2013.15 23419383

[20] Nam SY, Kim BC, Ryu KH, Park BJ. Prevalence and risk factors of irritable bowel syndrome in healthy screenee undergoing colonoscopy and laboratory tests. J Neurogastroenterol Motil. 2010;16: 47–51. 10.5056/jnm.2010.16.1.47 20535326 PMC2879825

[21] Goodpaster BH. Measuring body fat distribution and content in humans. Curr Opin Clin Nutr Metab Care. 2002;5: 481–487. 12172470 10.1097/00075197-200209000-00005

[22] Thaete FL, Colberg SR, Burke T, Kelley DE. Reproducibility of computed tomography measurement of visceral adipose tissue area. Int J Obes Relat Metab Disord. 1995;19: 464–467. 8520635

[23] Hoddinott P, Morgan H, MacLennan G, Sewel K, Thomson G, Bauld L, et al Public acceptability of financial incentives for smoking cessation in pregnancy and breast feeding: A survey of the british public. BMJ Open. 2014;4: e005524-2014-005524. 10.1136/bmjopen-2014-005524 PMC4120368 25037645

[24] Reding KW, Cain KC, Jarrett ME, Eugenio MD, Heitkemper MM. Relationship between patterns of alcohol consumption and gastrointestinal symptoms among patients with irritable bowel syndrome. Am J Gastroenterol. 2013;108: 270–276. 10.1038/ajg.2012.414 23295280 PMC3697482

[25] Kubo M, Fujiwara Y, Shiba M, Kohata Y, Yamagami H, Tanigawa T, et al. Differences between risk factors among irritable bowel syndrome subtypes in apanese adults. Neurogastroenterol Motil. 2011;23: 249–254. 10.1111/j.1365-2982.2010.01640.x 21122032

[26] Koch CA, Uwaifo GI. Are gastrointestinal symptoms related to diabetes mellitus and glycemic control? Eur J Gastroenterol Hepatol. 2008;20: 822–825. 10.1097/MEG.0b013e3282f5f75e 18794593

[27] Sim J, Wright CC. The kappa statistic in reliability studies: Use, interpretation, and sample size requirements. Phys Ther. 2005;85: 257–268. 15733050

[28] Zhao YF, Guo XJ, Zhang ZS, Ma XQ, Wang R, Yan XY, et al Epidemiology of functional diarrhea and comparison with diarrhea-predominant irritable bowel syndrome: A population-based survey in china. PloS One. 2012;7: e43749 10.1371/journal.pone.0043749 22937091 PMC3427143

[29] Shoelson SE, Herrero L, Naaz A. Obesity, inflammation, and insulin resistance. Gastroenterology. 2007;132: 2169–2180. 17498510 10.1053/j.gastro.2007.03.059

[30] Talley NJ, Howell S, Poulton R. Obesity and chronic gastrointestinal tract symptoms in young adults: A birth cohort study. Am J Gastroenterol. 2004;99: 1807–1814. 15330923 10.1111/j.1572-0241.2004.30388.x

[31] Farzaneh N, Ghobaklou M, Moghimi-Dehkordi B, Naderi N, Fadai F. Effects of demographic factors, body mass index, alcohol drinking and smoking habits on irritable bowel syndrome: A case control study. Ann Med Health Sci Res. 2013;3: 391–396. 10.4103/2141-9248.117958 24116320 PMC3793446

[32] Byrt T, Bishop J, Carlin JB. Bias, prevalence and kappa. J Clin Epidemiol. 1993;46: 423–429. 8501467 10.1016/0895-4356(93)90018-v

[33] Burton C, McGorm K, Weller D, Sharpe M. Depression and anxiety in patients repeatedly referred to secondary care with medically unexplained symptoms: A case-control study. Psychol Med. 2011;41: 555–563. 10.1017/S0033291710001017 21272387

[34] Henningsen P, Herzog W. Irritable bowel syndrome and somatoform disorders. J Psychosom Res. 2008;64: 625–629. 10.1016/j.jpsychores.2008.02.015 18501264

[35] Collins SM. A role for the gut microbiota in IBS. Nat Rev Gastroenterol Hepatol. 2014;11: 497–505. 10.1038/nrgastro.2014.40 24751910

[36] Goebel-Stengel M, Stengel A, Schmidtmann M, Voort I, Kobelt P, Monnikes H. Unclear abdominal discomfort: Pivotal role of carbohydrate malabsorption. J Neurogastroenterol Motil. 2014;20: 228–235. 10.5056/jnm.2014.20.2.228 24840375 PMC4015196

[37] Campbell AK, Matthews SB, Vassel N, Cox CD, Naseem R, Chaichi J, et al Bacterial metabolic ‘toxins’: A new mechanism for lactose and food intolerance, and irritable bowel syndrome. Toxicology. 2010;278: 268–276. 10.1016/j.tox.2010.09.001 20851732

[38] Heizer WD, Southern S, McGovern S. The role of diet in symptoms of irritable bowel syndrome in adults: A narrative review. J Am Diet Assoc. 2009;109: 1204–1214. 10.1016/j.jada.2009.04.012 19559137

